# Aminoglycosides use has a risk of acute kidney injury in patients without prior chronic kidney disease

**DOI:** 10.1038/s41598-022-21074-x

**Published:** 2022-10-14

**Authors:** Chu-Lin Chou, Nai-Chen Chuang, Hui-Wen Chiu, Chia-Te Liao, Yung-Ho Hsu, Tzu-Hao Chang

**Affiliations:** 1 Division of Nephrology, Department of Internal Medicine, School of Medicine, College of Medicine, Taipei Medical University, No. 250, Wuxing Street, Xinyi District, Taipei City, 110 Taiwan, ROC; 2 Taipei Medical University-Research Center of Urology and Kidney, Taipei Medical University, Taipei, Taiwan, ROC; 3 Division of Nephrology, Department of Internal Medicine, Shuang Ho Hospital, Taipei Medical University, New Taipei City, Taiwan, ROC; 4 Division of Nephrology, Department of Internal Medicine, Hsin Kuo Min Hospital, Taipei Medical University, Taoyuan City, Taiwan, ROC; 5 Clinical Data Center, Office of Data Science, Taipei Medical University, Taipei City, Taiwan, ROC; 6 Graduate Institute of Clinical Medicine, College of Medicine, Taipei Medical University, Taipei, Taiwan, ROC; 7 Department of Medical Research, Shuang Ho Hospital, Taipei Medical University, New Taipei City, Taiwan, ROC; 8 Graduate Institute of Biomedical Informatics, College of Medical Science and Technology, International Center for Health Information Technology, Taipei Medical University, 11F., Sec. 2, Keelung Rd., Da’an Dist., Taipei City, 106 Taiwan, ROC; 9 Clinical Big Data Research Center, Taipei Medical University Hospital, Taipei City, Taiwan, ROC

**Keywords:** Nephrology, Kidney diseases, Acute kidney injury

## Abstract

The outcome of acute kidney injury (AKI) as a result of aminoglycosides (AGs) use remains uncertain in patients without prior chronic kidney disease (CKD). Therefore, we explored the outcomes of AGs use on AKI episodes associated with renal recovery and progress in patients without prior CKD in Taiwan. This was a retrospective cohort study by using the Taipei Medical University Research Database from January 2008 to December 2019. 43,259 individuals without CKD who had received parenteral AGs were enrolled. The exposed and unexposed groups underwent propensity score matching for age, gender, patients in intensive care unit/emergency admission, and covariates, except serum hemoglobin and albumin levels. We identified an exposed group of 40,547 patients who used AGs (median age, 54.4 years; 44.3% male) and an unexposed group of 40,547 patients without AG use (median age, 55.7 years; 45.5% male). There was the risk for AKI stage 1 (adjusted hazard ratio [HR] 1.34; 95% confidence interval [CI] 1.00–1.79; *p* = 0.05) in patients that used AGs in comparison with the control subjects. Moreover, patients using AGs were significantly associated neither with the progression to acute kidney disease (AKD) stages nor with the progression to end-stage renal disease (ESRD) on dialysis. Further analyzed, there was an increased risk of AKI episodes for serum albumin levels less than 3.0 g/dL and hemoglobin levels less than 11.6 g/dL. Among patients without prior CKD, AGs-used individuals were associated with AKI risks, especially those at relatively low albumin (< 3.0 g/dL) or low hemoglobin (< 11.6 g/dL). That could raise awareness of AGs prescription in those patients in clinical practice.

## Introduction

Aminoglycosides (AGs), one of the oldest antibiotics, have been used since the 1940s to treat severe infections caused primarily by Gram-negative bacteria^1^. Since then, gentamycin, amikacin, and tobramycin have been the most commonly used parenteral AGs, typically reserved for complicated and multidrug-resistant infections^2^. AGs are concentration-dependent. They have a bactericidal activity that inhibits protein synthesis in the 30S subunit of the bacterial ribosome, leading to an interruption of bacterial protein synthesis, ultimately leading to bacterial death^3,4^. Because of the increasing rates of multidrug-resistant infections to quinolones and broad-spectrum β-lactams, AGs are still prescribed, and their frequency of administration needs to be described precisely^5^.

AGs are a leading source of acute kidney injury (AKI)^6^. AGs use could either evoke renal vasoconstriction^7^ or accumulate in proximal tubular cells, probably leading to tubular epithelial cell necrosis^8^. The incidence of AGs-induced AKI is reported to be approximately 5–25%^9,10^, and non-oliguric AKI typically occurs after 5–7 days of treatment^11^. Thus, identifying the possible risk factors for AGs-associated nephrotoxicity is crucial to reducing kidney injury and ensuing complications in patients that use AGs. The risk factors for AKI have been reported to include older age, diabetes, chronic kidney disease (CKD), liver disease, iodinated contrast, nephrotoxic antibiotics, sepsis, congestive heart failure, hypoalbuminemia, and hypotension^10–15^.

Over decades, increasing attention has been focused on subsequent kidney outcomes, including acute kidney disease (AKD) and end-stage renal disease (ESRD), following AGs-induced AKI^16^. Previously reported, our studies have shown that gentamicin evoked the release of cytochrome C from the mitochondria to trigger caspase-3 and consequently cause apoptosis in renal tubular cells^12,17,18^. Although irreversible kidney damage seemed uncommon in animals^19^, an absence of renal recovery has been observed in humans. Clinical outcomes of renal recovery following AGs-induced AKI episodes remain unknown and are supported by limited clinical data. Therefore, we sought to determine risk factors for AGs-induced AKI episodes and the outcome of renal recovery or progression to AKD and ESRD on dialysis in these patients using AGs.

There is little evidence of the renal outcome in patients without CKD and who use AGs. Therefore, in this study, using the Taipei Medical University Research Database (TMURD), we explored the effect of AGs use on the outcomes of kidney recovery and a continuum that includes AKI, AKD, and ESRD on dialysis, in those patients without prior CKD in Taiwan.

## Methods

### Study population

The study was based on data from the TMURD, which contains the electronic health records of more than 3 million patients from 3 affiliated teaching hospitals: Taipei Medical University Hospital (TMUH), Wan Fang Hospital (WFH), and Shuang Ho Hospital (SHH). Informed consent is waived due to personal information that had been de-identified in the TMURD and informed consent waiver was approved by the Institutional Review Board of the Taipei Medical University (TMU-JIRB-N201912034). All information allowing an enrolled patient to be identified was encrypted. This study was carried out in accordance with the approved protocol and the Declaration of Helsinki. All experimental protocols were approved by the Institutional Review Board of the Taipei Medical University, Taipei, Taiwan. A request for the analytic methods should be sent to the corresponding author.

**Figure 1:**
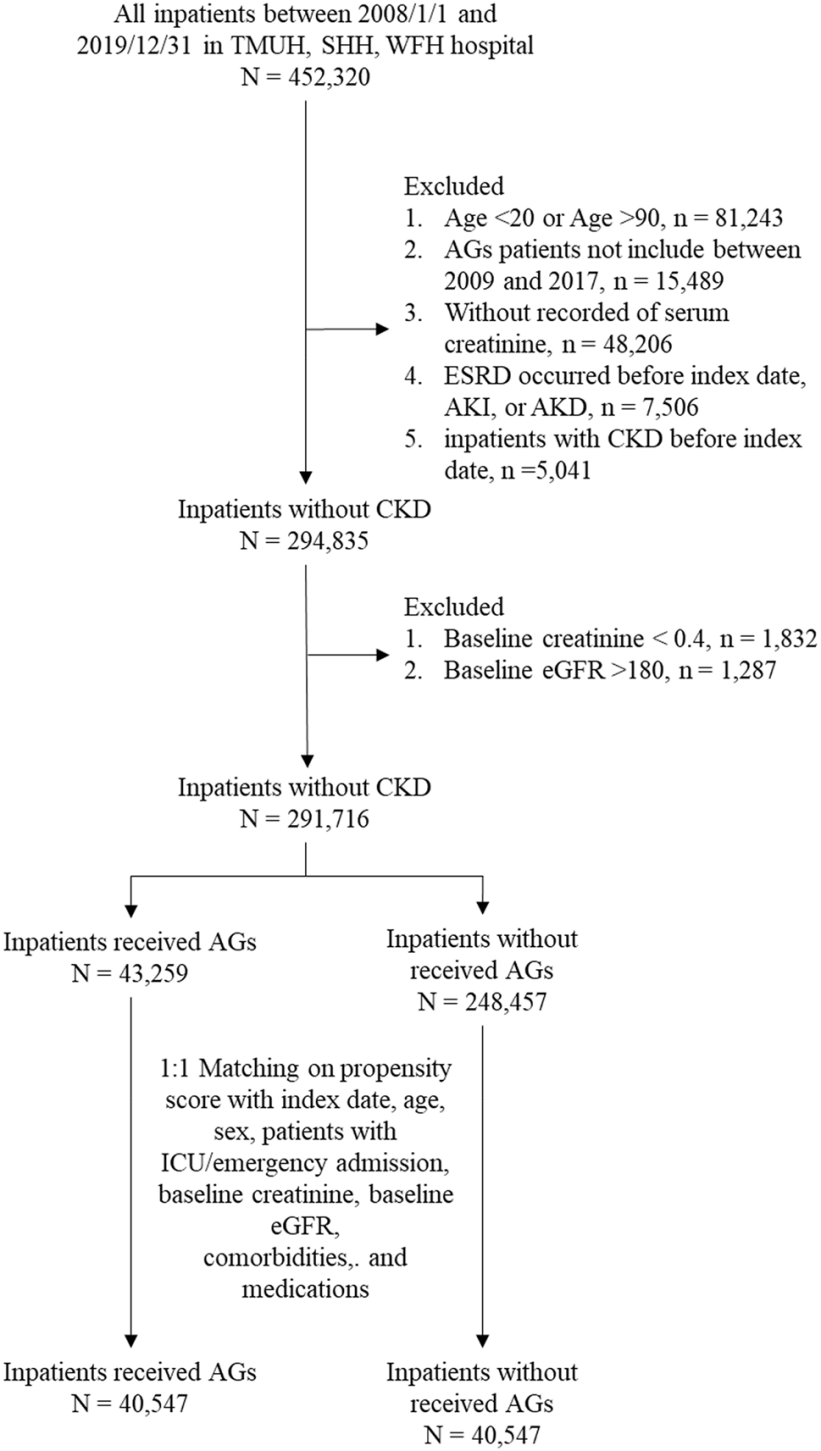
Flow chart of the study population. AGs, aminoglycosides; AKI, acute kidney injury; AKD, acute kidney disease; CKD, chronic kidney disease; ESRD, end-stage renal disease; eGFR, estimated glomerular filtration rate; SHH, Shuang Ho hospital; TMUH, Taipei Medical University hospital; WFH, Wan Fang hospital.

We enrolled 452,320 inpatients from 2008 to 2019 in TMUH, WFH, and SHH. Our exclusion criteria were age out of the 20- to 90-year range (n = 81,243) and reception of AGs out of the 2009–2017 period (n = 15,489). All patients without recorded values for serum creatinine (n = 48,206), occurring ESRD (n = 7506), or CKD (n = 5041) before index date were excluded. Moreover, baseline creatinine under 0.4 mg/dL (n = 1832), and estimated glomerular filtration rate (eGFR) over 180 mL/min/1.73 m^2^ (n = 1287) on patients' serum data were also excluded. The study included 291,716 patients who were divided into patients who received AGs (n = 43,259) and the ones who did not receive AGs (n = 248,457). Because of the imbalance in number and baseline characteristics between the 2 groups, we used propensity score matching (PSM) to match patients taking and not taking AGs. The final study included 40,547 patients who took AGs and 40,547 patients who did not take AGs (Fig. 1).

### Demographic variables

Inpatients who were on parenteral AGs (gentamicin, amikacin, and tobramycin) after admission were assigned to the exposed group, whereas those who did not receive parenteral AGs constituted the unexposed group. The index date was defined as the date on which inpatients first received AGs (exposed group) or the date of admission (unexposed group) between January 1, 2008, and December 31, 2009. The definitions of baseline serum, including creatinine, eGFR, hemoglobin (HGB), and ALB, are as follows: baseline creatinine was defined as the creatinine level within 7 days before the index date; baseline eGFR and HGB were defined as the respective values within 7 days after the index date; baseline ALB was defined as the value within 3 months before the index date. Baseline HGB and ALB were also divided into high/low HGB (≥ 13 g/dL/ < 13 g/dL) and ALB (≥ 3.5 g/dL/ < 3.5 g/dL) groups. According to these studies^20,21^, the definitions of anemia and hypoalbuminemia were HGB < 13 g/dL and ALB < 3.5 g/dL, respectively. The following comorbidities 1 year before the index date were recorded as confounding factors (CKD, hypertension, diabetes mellitus, hyperlipidemia, acute myocardial infarction, ischemic stroke, peripheral artery disease (PAD), chronic obstructive pulmonary disease (COPD), and chronic liver disease (CLD)). Patients who received medications, including angiotensin-converting enzyme inhibitors/angiotensin-receptor blockers (ACEIs/ARBs), beta-2 blockers, calcium channel blockers (CCBs), sulfonylureas, metformin, insulin, statin, antiplatelet, non-steroidal anti-inflammatory drugs (NSAIDs), and ccontrast media drugs, were all defined as patients who had receiving medications 1 year before the index date. All the disease diagnosis codes according to ICD-9-CM, ICD-10-CM, NHI procedure code and ATC classification of medications please see Supplemental Table S1.

### Study endpoint

Our primary endpoints, which represented AKI-AKD-ESRD progression, were defined from ADQI^22^ and KDIGO workshop^23^. AKI was defined as an abrupt decrease in kidney function occurring within 7 days or less after the index date, divided into AKI stages 0, 1, 2, and 3 multiplied by serum creatinine (SCr) levels. AKI stage 0 (SCr < 1.5 ×), AKI stage 1 (SCr 1.5 × –1.9 ×), AKI stage 2 (SCr 2.0 × –2.9 ×), and AKI stage 3 (SCr 3.0 × +). AKD was described as acute or subacute damage and loss of kidney function for a duration of between 7 and 90 days after exposure to an AKI episode and is divided into AKD stages 0, 1, 2, and 3 multiplied by SCr levels: AKD stage 0 (SCr < 1.5 ×), AKD stage 1 (SCr 1.5 × –1.9 ×), AKD stage 2 (SCr 2.0 × –2.9 ×), and AKD stage 3 (SCr 3.0 × +). All the SCr levels were chosen using the highest value during 0–7 and 7–90 days. ESRD on dialysis was defined as an order code by National Health Insurance, including 58001C, 58019C, 58020C, 58021C, 58022C, 58023C, 58024C, 58025C, 58029C, 58002C, 58009A, 58009B, 58010A, 58010B, 58011A, 58011AB, 58011B, 58011C, 58012A, 58012B, 58017B, 58017C, 58026C, 58028C.

### Statistical analysis

Descriptive statistics were used to summarize the demographic data. Continuous variables are presented as the mean with standard deviation and the median with interquartile range (IQR), whereas categorical variables are presented as the number of enrollees and percentage (%). The standardized difference was a difference in means or proportions divided by the standard error and imbalance defined as an absolute value greater than 0.10. Considering that the number of participants in the exposed group was substantially smaller than that in the unexposed group, we chose a greedy and nearest-neighbor matching for the PSM algorithm. We conducted 1:1 PSM with age, sex, patients in intensive care unit/emergency admission (ICU/ED), baseline creatinine, baseline eGFR, comorbidities, and medication use to reduce selection bias between the 2 groups. The Cox proportional hazards regression model was used to evaluate the association between receiving AGs, serum marker values, and renal outcomes after controlling for demographic and clinical factors. Renal outcomes, including AKI stage, AKD stage, and ESRD, were treated as the events. Death or loss to follow-up or the end of the follow-up period as the censoring indicator. Subgroup analysis was used to evaluate the association between ALB, HGB, and renal outcomes in patients who were and were not taking AGs, respectively. Further, a restricted cubic spline curve with knots placed at the 5th, 25th, 50th, 75th, and 95th percentiles were used to examine the association between HGB, ALB, and risk for AKI events. A 2-sided statistical test at 5% significance was used. Analyses were performed using SAS (version 7.11; SAS Institute, Cary, NC, USA).

## Results

### Cohort

A total of 291,716 participants were enrolled and divided into patients not taking AGs (n = 248,457) and patients taking AGs (n = 43,259) from January 2009 to December 2017. Patients and demographic characteristics of the 2 groups are listed in Table 1. Before matching, the median age with IQR was 55.8 (41.4, 68.8) years and 54.9 (41.3, 68.3) years in the 2 groups. Male patients not taking AGs were more numerous than patients taking AGs (52.3% vs. 43.9%, the absolute standardized difference [ASD]: 0.169). According to baseline serum creatinine (0.8 (0.7, 1.0) vs. 0.8 (0.7, 1.0) mg/dL, ASD: 0.143) was significantly higher in patients not taking AGs than in patients taking AGs. The low HGB group had significantly more patients taking AGs than patients not taking AGs (65.8% vs. 37.8%, ASD: 0.585, Table 1). Patients with acute myocardial infarction had significantly more patients not taking AGs than patients taking AGs (1.5% vs. 0.4%, ASD: 0.109, Table 1). As for medications used, there were significantly more patients who used beta-2 blockers (13.6% vs. 8.7%, ASD: 0.158), CCB (23.9% vs. 11.8%, ASD: 0.321), sulfonylureas (4.6% vs. 2.6%, ASD: 0.106), metformin (6.5% vs. 4.0%, ASD: 0.112), insulin (16.5% vs. 10.0%, ASD: 0.194), and NSAIDs (48.4% vs. 13.2%, ASD: 0.824) taking AGs than those not taking AGs. After PSM, there were no significant differences in variables between the 2 groups, except for unmatched variables. As for the low HGB group, the percentage was still significantly higher in patients taking AGs than in patients not taking AGs (65.1% vs. 39.9%, ASD: 0.522, Table 1). The percentage of patients taking AGs in the low ALB group was significantly higher than that of patients not taking AGs (45.1% vs. 34.8%, ASD: 0.211, Table 1). The number and percentage of outcome events (AKI, AKD, ESRD) before and after PSM were provided in Supplemental Table S2.

**Table 1:** Demographic and clinical characteristics of patients without prior CKD with and without using AGs.

	Before matching				After matching			
	Total	Without AGs	With AGs	ASD	Total	Without AGs	With AGs	ASD
	n = 291,716	n = 248,457	n = 43,259		n = 81,094	n = 40,547	n = 40,547	
Age	55.6 (41.3, 68.4)	55.8 (41.4, 68.4)	54.9 (41.3, 68.3)	0.001	55.1 (41.1, 68.1)	55.7 (41.5, 68.3)	54.4 (40.8, 67.9)	0.018
Sex, male	148,855 (51.0)	129,885 (52.3)	18,970 (43.9)	**0.169**	36,429 (44.9)	18,461 (45.5)	17,968 (44.3)	0.024
Patients in ICU/ED	81,440 (27.9)	69,732 (28.1)	11,708 (27.1)	0.022	22,549 (27.8)	11,611 (28.6)	10,938 (27.0)	0.037
**Serum**								
Baseline Creatinine, mg/dL	0.8 (0.7, 1.0)	0.8 (0.7, 1.0)	0.8 (0.7, 1.0)	**0.143**	0.8 (0.7, 1.0)	0.8 (0.7, 1.0)	0.8 (0.7, 1.0)	0.008
Baseline eGFR, mL/min/1.73 m^2^	86.1 (68.4, 104.4)	86.2 (68.3, 104.3)	85.5 (68.8, 104.9)	0.050	86.6 (69.4, 105.4)	87.3 (69.7, 105.8)	85.7 (69.1, 105.1)	0.007
Baseline HGB group, < 13 g/dL	106,021 (41.1)	85,838 (37.8)	20,183 (65.8)	**0.585**	33,342 (51.0)	14,622 (39.9)	18,720 (65.1)	**0.522**
Baseline ALB group, < 3.5 mg/dL	11,123 (37.5)	8539 (35.7)	2584 (44.8)	**0.186**	3419 (41.1)	1114 (34.8)	2305 (45.1)	**0.211**
**Comorbidities**								
Hypertension	56,975 (19.5)	47,598 (19.2)	9377 (21.7)	0.063	16,808 (20.7)	8411 (20.7)	8397 (20.7)	0.001
Diabetes mellitus	29,865 (10.2)	24,690 (9.9)	5175 (12.0)	0.065	9150 (11.3)	4603 (11.4)	4547 (11.2)	0.004
Hyperlipidemia	26,446 (9.1)	22,519 (9.1)	3927 (9.1)	0.001	7262 (9.0)	3639 (9.0)	3623 (8.9)	0.001
Acute myocardial infarction	3840 (1.3)	3658 (1.5)	182 (0.4)	**0.109**	351 (0.4)	172 (0.4)	179 (0.4)	0.003
Ischemic stroke	13,742 (4.7)	11,883 (4.8)	1859 (4.3)	0.023	3313 (4.1)	1639 (4.0)	1674 (4.1)	0.004
PAD	1676 (0.6)	1420 (0.6)	256 (0.6)	0.003	498 (0.6)	261 (0.6)	237 (0.6)	0.008
COPD	13,551 (4.7)	11,402 (4.6)	2149 (5.0)	0.018	3824 (4.7)	1857 (4.6)	1967 (4.9)	0.013
CLD	8718 (3.0)	7402 (3.0)	1316 (3.0)	0.004	2357 (2.9)	1173 (2.9)	1184 (2.9)	0.002
**Medication**								
ACEI/ARB	32,444 (11.1)	26,925 (10.8)	5519 (12.8)	0.060	9544 (11.8)	4764 (11.8)	4780 (11.8)	0.001
Beta-2 blocker	27,372 (9.4)	21,483 (8.7)	5889 (13.6)	**0.158**	9525 (11.8)	4744 (11.7)	4781 (11.8)	0.003
CCB	39,519 (13.6)	29,197 (11.8)	10,322 (23.9)	**0.321**	16,384 (20.2)	8080 (19.9)	8304 (20.5)	0.014
Sulfonylureas	8418 (2.9)	6444 (2.6)	1974 (4.6)	**0.106**	3246 (4.0)	1632 (4.0)	1614 (4.0)	0.002
Metformin	12,770 (4.4)	9957 (4.0)	2813 (6.5)	**0.112**	4755 (5.9)	2431 (6.0)	2324 (5.7)	0.011
Insulin	31,979 (11.0)	24,827 (10.0)	7152 (16.5)	**0.194**	11,661 (14.4)	5829 (14.4)	5832 (14.4)	< 0.001
Statin	16,994 (5.8)	14,553 (5.9)	2441 (5.6)	0.009	4428 (5.5)	2210 (5.5)	2218 (5.5)	0.001
Antiplatelet drug	30,720 (10.5)	27,211 (11.0)	3509 (8.1)	0.097	6437 (7.9)	3144 (7.8)	3293 (8.1)	0.014
NSAIDs	53,861 (18.5)	32,907 (13.2)	20,954 (48.4)	**0.824**	36,958 (45.6)	18,667 (46.0)	18,291 (45.1)	0.019
Contrast media	35,892 (12.3)	30,380 (12.2)	5512 (12.7)	0.016	8978 (11.1)	4422 (10.9)	4556 (11.2)	0.011
Propensity score	0.9 ± 0.1	0.9 ± 0.1	0.7 ± 0.2	**1.429**	0.7 ± 0.2	0.7 ± 0.2	0.7 ± 0.2	0.005

Data are presented as mean with standard deviation and median with inter-quartiles for continuous variables; number of subjects with percentage (%) for categorical variables.

Absolute standardized difference, difference in means or proportions divided by standard error; imbalance defined as absolute value greater than 0.10.

1:1 Matching on propensity score with index date, age, sex, patients in ICU/ED, baseline creatinine, eGFR, comorbidities, and medications.

AGs, aminoglycosides (including parenteral gentamicin, amikacin, and tobramycin); ACEI, angiotensin-converting enzyme inhibitor; ARB, angiotensin receptor blocker; CCB, calcium channel blockers; CLD, chronic liver disease; COPD, chronic obstructive pulmonary disease; ICU/ED, intensive care unit/emergency admission; PAD, peripheral artery disease; NSAIDs, Non-steroidal anti-inflammatory drugs; ASD, Absolute standardized difference.

Boldface is presented as statistically significant difference (standardized difference > |0.1|).

### Group and subgroup analyses

The proportion of AKI in patients taking AGs use in our cohort was 1.25%. The crude hazard ratio (HR) and adjusted HR (aHR) for kidney outcomes in patients taking and not taking AGs are shown in Table 2. Compared with that for patients not taking AGs, the HR for progression to AKI stage 1 was 1.54 (95% confidence interval [CI] 1.30–1.84, *p* < 0.001) for patients taking AGs. After adjustments (for the baseline HGB group and the baseline ALB group), patients taking AGs had a higher risk of progression to AKI stage 1 (aHR: 1.34, 95% CI 1.00–1.79, *p* = 0.05). However, there was no significant risk for progression to AKI stage 2 (aHR: 0.99, 95% CI 0.66–1.47, *p* = 0.954) and AKI stage 3 (aHR: 0.85, 95% CI 0.49–1.47, *p* = 0.560). Moreover, the patients taking AGs were significantly associated neither with progression to all AKD stages nor with progression to ESRD on dialysis (Table 2). In addition, patients with AKI stage 2 were at risk of progression to AKD stage 3 (aHR: 2.88, 95% CI 1.42–5.85, *p* = 0.003, S1 Table). Also, patients with AKI stage 3 were at risk of progression to AKD stage 3 (aHR: 8.01, 95% CI 4.08–15.71, *p* < 0.001, Supplemental Table S3).

**Table 2:** A hazard ratio of kidney outcome (AKI, AKD, and ESRD on dialysis) in patients without prior CKD with using AGs compared with using non-AGs in the Cox model.

Event, n (%)	Without AGs	With AGs	HR (95% CI)	*p* value	aHR (95% CI)	*p* value
Baseline patients	n = 40,547	n = 40,547				
AKI stage (0, 1)*	206 (0.5)	318 (0.8)	1.54 (1.30, 1.84)	**< 0.001**	1.34 (1.00, 1.79)	**0.050**
AKI stage (0, 2)*	114 (0.3)	126 (0.3)	1.11 (0.86, 1.43)	0.426	0.99 (0.66, 1.47)	0.954
AKI stage (0, 3)*	67 (0.2)	63 (0.2)	0.94 (0.67, 1.33)	0.739	0.85 (0.49, 1.47)	0.560

Patients with AKI	n = 387	n = 507				
AKD stage (0, 1)^†^	39 (10.1)	63 (12.4)	1.25 (0.84, 1.87)	0.272	1.46 (0.78, 2.72)	0.236
AKD stage (0, 2)^†^	33 (8.5)	45 (8.9)	1.07 (0.68, 1.67)	0.781	0.88 (0.42, 1.84)	0.738
AKD stage (0, 3)^†^	32 (8.3)	53 (10.5)	1.29 (0.83, 1.99)	0.263	1.47 (0.82, 2.61)	0.196

Patients with AKI and AKD	n = 104	n = 161				
ESRD on dialysis^§^	4 (3.9)	9 (5.6)	1.43 (0.44, 4.65)	0.551	NA	NA

AGs, aminoglycosides (parenteral gentamicin, amikacin, and tobramycin); AKI, acute kidney injury; AKD, acute kidney disease; ESRD, end-stage renal disease; HR, hazard ratio; aHR, adjust hazard ratio; NA, not available due to small sample size.

*Model was adjusted by baseline hemoglobin group and baseline albumin group.

^†^Model was adjusted by baseline hemoglobin group, baseline albumin group and AKI stage.

^§^Model was adjusted by baseline hemoglobin group, baseline albumin group, AKI stage and AKD stage.

Table 3 shows the association between anemia (HGB < 13 g/dL), hypoalbuminemia (ALB < 3.5 g/dL), and kidney outcomes in the Cox proportional hazards regression model. The results show that hypoalbuminemia was a risk factor for progression to AKI stage 1 (aHR: 2.73, 95% CI 2.04–3.56, *p* < 0.001), AKI stage 2 (aHR: 2.90, 95% CI 1.91–4.41, *p* < 0.001), and AKI stage 3 (aHR: 3.04, 95% CI 1.71–5.39, *p* < 0.001) in the whole population. In subgroup analyses for patients taking AGs, the results still show that hypoalbuminemia was a risk factor for progression to AKI stages (AKI stage 1: aHR: 2.33, 95% CI 1.64–3.29, *p* < 0.001; AKI stage 2: aHR: 1.93, 95% CI 1.16–3.22, *p* = 0.012). For patients not taking AGs, hypoalbuminemia was a risk factor for progression to AKI stage 1 (aHR: 3.71, 95% CI 2.21–6.21, *p* < 0.001), AKI stage 2 (aHR: 5.55, 95% CI 2.76–11.14, *p* < 0.001), and AKI stage 3 (aHR: 5.18, 95% CI 2.16–12.45, *p* < 0.001). In addition, anemia was a risk factor for progression to AKD stage 1 (aHR: 2.35, 95% CI 1.09–5.06, *p* = 0.029) in the whole population after adjustments. Moreover, AKI stage 2 (aHR: 4.35, 95% CI 1.93–9.81, *p* < 0.001) and AKI stage 3 (aHR: 7.45, 95% CI 3.07–18.07, *p* < 0.001) were risk factors for progression to AKD stage 3 in patients taking AGs (Supplemental Table S4); AKI stage 3 (aHR: 8.19, 95% CI 2.79–24.02, *p* < 0.001) was a risk factor for progression to AKD stage 3 in patients not taking AGs (Supplemental Table S5).

**Table 3:** A hazard ratio of anemia (HGB < 13 g/dL) and hypoalbuminemia (ALB < 3.5 g/dL) on kidney outcomes (AKI, AKD, and ESRD on dialysis) in patients without prior CKD with and without using AGs in the Cox model.

Outcome	Serum group	Model 1, total		Model 2, with AGs		Model 3, without AGs	
		aHR (95% CI)	*p* value	aHR (95% CI)	*p* value	aHR (95% CI)	*p* value
**AKI stage (0, 1)**^a,b^							
	Baseline HGB < 13 g/dL	0.94 (0.69, 1.29)	0.713	0.83 (0.56, 1.23)	0.359	1.10 (0.66, 1.86)	0.714
	Baseline ALB < 3.5 mg/dL	2.73 (2.04, 3.65)	**< 0.001**	2.33 (1.64, 3.29)	**< 0.001**	3.71 (2.21, 6.21)	**< 0.001**
**AKI stage (0, 2)**^a,b^							
	Baseline HGB < 13 g/dL	0.86 (0.55, 1.34)	0.506	0.94 (0.52, 1.72)	0.849	0.72 (0.38, 1.36)	0.310
	Baseline ALB < 3.5 mg/dL	2.90 (1.91, 4.41)	**< 0.001**	1.93 (1.16, 3.22)	**0.012**	5.55 (2.76, 11.14)	**< 0.001**
**AKI stage (0, 3)**^a,b^							
	Baseline HGB < 13 g/dL	0.55 (0.31, 0.97)	**0.040**	0.54 (0.25, 1.16)	0.114	0.54 (0.24, 1.22)	0.140
	Baseline ALB < 3.5 mg/dL	3.04 (1.71, 5.39)	**< 0.001**	1.95 (0.93, 4.10)	0.077	5.18 (2.16, 12.45)	**< 0.001**
**AKD stage (0, 1)**^c,d^							
	Baseline HGB < 13 g/dL	2.35 (1.09, 5.06)	**0.029**	2.31 (0.89, 6.01)	0.086	2.44 (0.67, 8.81)	0.174
	Baseline ALB < 3.5 mg/dL	1.12 (0.62, 2.01)	0.713	1.17 (0.58, 2.34)	0.666	1.00 (0.33, 3.02)	0.995
**AKD stage (0, 2)**^c,d^							
	Baseline HGB < 13 g/dL	2.23 (0.85, 5.90)	0.105	2.74 (0.62, 12.00)	0.182	1.73 (0.46, 6.51)	0.417
	Baseline ALB < 3.5 mg/dL	2.20 (0.89, 5.40)	0.086	1.85 (0.60, 5.64)	0.282	2.78 (0.61, 12.76)	0.189
**AKD stage (0, 3)**^c,d^							
	Baseline HGB < 13 g/dL	0.97 (0.54, 1.73)	0.912	1.43 (0.64, 3.19)	0.379	0.44 (0.17, 1.14)	0.092
	Baseline ALB < 3.5 mg/dL	1.15 (0.65, 2.04)	0.625	1.07 (0.53, 2.14)	0.855	1.43 (0.53, 3.89)	0.480
**ESRD on dialysis**^e,f^							
	Baseline HGB < 13 g/dL	NA	NA	NA	NA	NA	NA
	Baseline ALB < 3.5 mg/dL	1.35 (0.15, 12.22)	0.788	1.35 (0.15, 12.22)	0.788	NA	NA

Model 1 was enrolled all the study patients; model 2 was enrolled patients with AGs and model 3 was enrolled with patients without AGs.

AGs, aminoglycosides (parenteral gentamicin, amikacin, and tobramycin); AKI, acute kidney injury; AKD, acute kidney disease; aHR, adjust hazard ratio; ALB, albumin; ESRD, end-stage renal disease; HGB, hemoglobin; NA, not available due to small sample size.

Significant values are in [Bold].

^a^Model 1 was adjusted by AGs, baseline hemoglobin group and baseline albumin group.

^b^Model 2 and model 3 were adjusted by baseline hemoglobin group and baseline albumin group.

^c^Model 1 was adjusted by AGs, baseline hemoglobin group, baseline albumin group and AKI stage.

^d^Model 2 and model 3 were adjusted by baseline hemoglobin group, baseline albumin group and AKI stage.

^e^Model 1 was adjusted by AGs, baseline hemoglobin group, baseline albumin group, AKI stage and AKD stage.

^f^Model 2 and model 3 were adjusted by baseline hemoglobin group, baseline albumin group, AKI stage and AKD stage.

**Figure 2:**
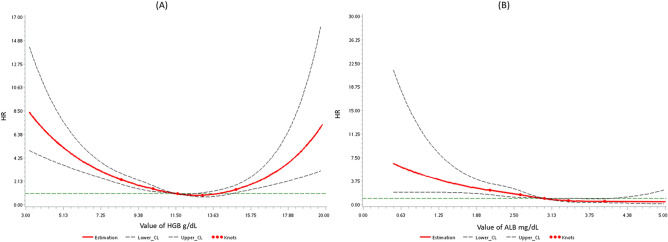
Association between (**A**) hemoglobin (HGB)/(**B**) albumin (ALB) and risk for AKI episodes in patients with aminoglycosides (AGs) use, as compared with non-AGs use, using restricted cubic splines with 5 knots located at 5th, 25th, 50th, 75th, 95th percentiles. Y-axis represents the adjusted hazard ratio for AKI event for any value of serum compared to individuals with 11.6 g/dL of HGB or 3 mg/dL ALB. Dash lines are 95 percent confidence intervals. Dots represent knots.

Figure 2 displays the association between serum levels of HGB and ALB and the risk of AKI episodes in patients taking AGs compared with those not taking AGs during the follow-up period. The risk of AKI episodes was higher in patients with serum HGB levels less than 11.6 g/dL (Fig. 2A). The patients with serum ALB levels less than 3.0 g/dL were more predisposed to AKI episodes (Fig. 2B). The more comprehensive 95% CIs were due to the smaller sample sizes as the values increased or decreased.

## Discussion

Our study explored the renal outcomes (AKI episodes with subsequent AKD and ESRD on dialysis) in patients taking and not taking AGs in Taiwan. Our findings suggested that AGs use is associated with a risk of AKI stage 1 episodes after multivariate adjustments and subgroup analyses. Moreover, patients taking AGs were significantly predisposed to either progression to all AKD stages or ESRD requiring dialysis. Hypoalbuminemia (ALB < 3.5 g/dL) was a risk factor for AKI stages 1–2. Anemia (HGB < 13 g/dL) was a risk factor for AKD stage 1. Further analyses revealed that the increased risk of AKI episodes occurs in serum ALB levels smaller than 3.0 g/dL and HGB levels smaller than 11.6 g/dL.

The safety and toxicity of AGs use remain still highlighted in clinical practice up to date. AGs use has been reported as one of the most common etiologies of AKI due to the nephrotoxicity of this class of medications^7,11^. Consequently, the Kidney Disease Improving Global Outcomes (KDIGO) AKI guidelines suggested the avoidance of AGs in patients at risk of (or suffering from) AKI; if possible, less nephrotoxic therapeutic alternatives to AGs should be used^24^. We found that AGs therapy is relatively risky to AKI episodes, a finding consistent with the results of other studies^9,10,25^. Of note, the incidence of AGs-induced AKI episodes was 1.07% in our study in Taiwan, which was lower than the values reported by previous studies (5–25%)^9,10^. Moreover, Ong et al. reported that AGs-induced AKI episodes are more common in the elderly, especially those receiving furosemide and those in shock more than in those taking only AGs^25^. The accumulation of AGs in the kidney could lead to AKI episodes, which are frequency dependent and increase with accumulated doses^26^. These consequences may explain the fact that more patients are experiencing a more accessible peak in AKI severity associated with AGs use.

Over the past years, more attention has been focused on renal outcomes following AKI episodes^10,16^. Limited clinical data have been available on renal function progression or recovery from AKI episodes following AGs therapy. In this study, we found no additional risk of AKD and ESRD on dialysis progression from AKI episodes, which indicated that those patients with AKI episodes had renal recovery follow AGs treatment. Oliguric renal failure with persistent nephrotoxicity is rare in elderly patients even if they were receiving once-daily AGs^27^. A retrospective observational study revealed no risk of chronic nephropathy with prolonged aminoglycoside exposure in patients with cystic fibrosis from 2006 to 2018^28^. Moreover, there was no association between AGs and the need for dialysis (adjusted OR = 0.83 [0.49–1.39], *p* = 0.477) in a prospective multicenter study of immunocompromised patients with bacterial pneumonia and septic shock^29^. In most patients who take AGs, persistent renal impairment is sporadic^30,31^. Reversible kidney function has been reported in AGs-induced AKI episodes^10,19,32^.

Recognizing the risk factors for AGs-induced nephrotoxic AKI and renal recovery are essential to reduce the number of AGs-induced AKI episodes and their associated complications. However, the factors associated with renal recovery in this population are unknown. Therefore, we determined the risk factors for AGs-induced AKI with renal recovery in patients treated with AGs in the TMURD. Our study identified ALB < 3.5 mg/dL as an independent risk factor for AKI episodes in patients taking AGs, unlike those not taking AGs. This result is consistent with those of previous studies^33–35^. To date, this clinical study investigated the association between serum ALB and HGB levels based on AGs-induced AKI episodes and renal recovery outcomes while adjusting for diuretic use and other leading risk factors. There is evidence supporting the association between low serum ALB levels and AGs-induced AKI episodes. Furthermore, reduced oncotic pressure with lower glomerular infiltration might aggravate the tubular injury associated with the use of AGs^36^. These phenomena highlight the importance of adequate oncotic pressure and glomerular filtration in controlling AGs-induced AKI episodes.

Anemia has been more frequently considered a factor associated with the severity of comorbid disease or poor disease prognosis. The impact of anemia has been reported for AKI episodes in anemic patients who use contrast media^20^, patients with preoperative and postoperative anemia^37,38^, and patients with critical illnesses^39^. In our study, we found that anemia (HGB < 13 g/dL) was a risk factor for AKD stage 1. Furthermore, HGB levels lower than 11.6 g/dL were associated with an increased risk of AKI episodes. The possible pathophysiology of AKI episodes supported that kidney hypoperfusion and hypoxia by anemia or vasoconstriction have been reported to cause kidney injury^40,41^.

The strengths of this study are the large sample size from the analysis of the TMURD, the consideration of the adjustment for primary risk factors, and the subgroup analysis. In addition, this study minimized the selection bias and confounding factors by matching the control group for age, sex, comorbidities, and medications, except serum HBG and ALB levels. Despite its strengths and novelty, this study has some limitations that should be mentioned. First, lifestyle and personal habits cannot be obtained from the TMU database. Second, given that this study was an observational study of drug epidemiology and not a randomized controlled trial, there may have been allocation bias and prescription bias. Although a randomized controlled trial is the best way to demonstrate pharmaceuticals' effects, most medical research uses drug epidemiology^42–44^. There are many situations where a randomized controlled trial is unsuitable, such as adverse effects, studying drug interactions, a genetic disposition to diseases, and examining drug overdose.

## Conclusion

This large cohort study suggested that AGs therapy could predispose to AKI episodes, especially in patients with low serum ALB (< 3.0 g/dL) and low HGB levels (< 11.6 g/dL). Moreover, the patients using AGs were not significantly associated with progression to AKD or ESRD. Thus, AGs use should be avoided in patients with hypoalbuminemia and anemia if possible.

## Supplementary Information

Supplementary Tables.

## Data Availability

The datasets collected and analyzed in our study are available from the corresponding author (Tzu-Hao Chang, email: kevinchang@tmu.edu.tw) on reasonable request.
