# Supplementary material for: Aminoglycosides use has a risk of acute kidney injury in patients without prior chronic kidney disease
Source: Sci Rep. 2022 Oct 14;12:17212. doi: 10.1038/s41598-022-21074-x (PMC9568559; doi:10.1038/s41598-022-21074-x)
Supplement: Supplementary file 1 — Supplementary Tables. [file 41598_2022_21074_MOESM1_ESM.docx]

**Supplemental Table S1 Disease diagnosis codes according to ICD-9-CM, ICD-10-CM, NHI procedure code and ATC classification of medications**

| Clinical outcomes or comorbidities | ICD-9-CM | ICD-10-CM |  |
| --- | --- | --- | --- |
| Hypertension | 401-405 | I10, I110, I119, I120, I129, I130, I1310, I1311, I132, I150, I151, I152, I158, I159, N262 |  |
| Diabetes | 250-250.3, 250.7, 250.4-250.6 | E0800, E0801, E08311, E08319, E08321, E08329, E08331, E08339, E08341, E08349, E08351, E08359, E0836, E0839, E0840, E0841, E0842, E0843, E0844, E0849, E0851, E0852, E0859, E08641, E0900, E0901, E09311, E09319, E09321, E09329, E09331, E09339, E09341, E09349, E09351, E09359, E0936, E0939, E0940, E0941, E0942, E0943, E0944, E0949, E0951, E0952, E0959, E09641, E1010, E1011, E1021, E1022, E1029, E10311, E10319, E1036, E1039, E1040, E1041, E1044, E1049, E1051, E1052, E1059, E10610, E10641, E1065, E1069, E109, E1100, E1101, E1121, E1122, E1129, E11311, E11319, E11321, E11329, E11331, E11339, E11341, E11349, E11351, E11359, E1136, E1139, E1140, E1141, E1142, E1143, E1144, E1149, E1151, E1152, E1159, E11641, E1165, E1169, E119, E1300, E1301, E1311, E1321, E1322, E1329, E13311, E13319, E13321, E13329, E13331, E13339, E13341, E13349, E13351, E13359, E1336, E1339, E1340, E1341, E1342, E1343, E1344, E1349, E1351, E1352, E1359, E13641, E139 |  |
| Hyperlipidemia | 272 | E780, E781, E782, E783, E784, E785 |  |
| Acute myocardial infarction | 410 | I22 |  |
| Ischemic stroke | 433, 434, 436 | I6300, I63011, I63012, I63019, I6302, I63031, I63032, I63039, I6309, I6310, I63111, I63112, I63119, I6312, I63131, I63132, I63139, I6319, I6320, I63211, I63212, I63219, I6322, I63231, I63232, I63239, I6329, I6330, I63311, I63312, I63319, I63321, I63322, I63329, I63331, I63332, I63339, I63341, I63342, I63349, I6339, I6340, I63411, I63412, I63419, I63421, I63422, I63429, I63431, I63432, I63439, I63441, I63442, I63449, I6349, I6350, I63511, I63512, I63519, I63521, I63522, I63529, I63531, I63532, I63539, I63541, I63542, I63549, I6359, I636, I638, I639, I6501, I6502, I6503, I6509, I651, I6521, I6522, I6523, I6529, I658, I659, I6601, I6602, I6603, I6609, I6611, I6612, I6613, I6619, I6621, I6622, I6623, I6629, I663, I668, I669, I6789 |  |
| PAD | 443.9, 441-441.9, 785.4, V43.4 | I7100, I7101, I7102, I7103, I711, I712, I713, I714, I715, I716, I718, I719, I739, I77819, I790, I96, Z95820, Z95828 |  |
| COPD | 490-496, 505, 506.4 | J40, J410, J411, J418, J42, J430, J431, J432, J438, J439, J440, J441, J449, J470, J471, J479 |  |
| CLD | 571.2, 571.4, 571.5, 571.6, 456-456.21, 572.2-572.8 | K702, K7030, K7031, K7210, K7211, K7290, K7291, K730, K731, K732, K738, K739, K740, K741, K742, K743, K744, K745, K7460, K7469, K754, K766, K767, K7681 |  |
| Medications | **ATC code** | |  |
| ACEI/ARB | C09 | |  |
| Beta-2 blocker | C07 | |  |
| CCB | C08 | |  |
| Sulfonylureas | A10BB | |  |
| Metformin | A10BA | |  |
| Insulin | A10A | |  |
| Statin | C10AA | |  |
| Antiplatelet | B01AC | |  |
| NSAID | M01A | |  |
| Contrast media | V08 | |  |

Abbreviation: ATC= Anatomical Therapeutic Chemical, ACEI/ARB= angiotensin-converting enzyme inhibitors/ angiotensin receptor blockers, CCB= calcium channel inhibitor, CLD= chronic liver disease, COPD= chronic obstructive pulmonary disease; ICD=International Classification of Diseases, PAD = peripheral artery disease, NSAIDs = Non-steroidal anti-inflammatory drugs

| **Supplemental Table S2 Events occurring at patients with or without AGs before and after matching** | | | | | |
| --- | --- | --- | --- | --- | --- |
|  | Before matching | |  | After matching all variables (except for matching HGB group, ALB group) | |
| Events, n (%) | Without AGs | With AGs |  | Without AGs | With AGs |
| Baseline population | n = 248,457 | n = 43,259 |  | n = 40,547 | n = 40,547 |
| AKI stage |  |  |  |  |  |
| 0 | 245937 (99.0) | 42669 (98.6) |  | 40160 (99.1) | 40040 (98.8) |
| 1 | 1410 (0.6) | 359 (0.8) |  | 206 (0.5) | 318 (0.8) |
| 2 | 684 (0.3) | 151 (0.4) |  | 114 (0.3) | 126 (0.3) |
| 3 | 426 (0.2) | 80 (0.2) |  | 67 (0.2) | 63 (0.2) |
| Patients with AKI | n = 2520 | n = 590 |  | n = 387 | n = 507 |
| AKD stage |  |  |  |  |  |
| 0 | 1797 (71.3) | 389 (65.9) |  | 283 (73.1) | 346 (68.2) |
| 1 | 280 (11.1) | 75 (12.7) |  | 39 (10.1) | 63 (12.4) |
| 2 | 208 (8.3) | 57 (9.7) |  | 33 (8.5) | 45 (8.9) |
| 3 | 235 (9.3) | 69 (11.7) |  | 32 (8.3) | 53 (10.5) |
| Patients with AKI and AKD | n = 723 | n = 201 |  | n = 104 | n = 161 |
| ESRD | 30 (4.2) | 10 (5.0) |  | 4 (3.9) | 9 (5.6) |

| **Supplemental Table S3** **A hazard ratio of AGs use on kidney outcome (AKI, AKD, and ESRD on dialysis) in patients without prior CKD in the Cox Model.** | | | | | |
| --- | --- | --- | --- | --- | --- |
|  | **HR (95% CI)** | ***P* value** |  | **aHR (95% CI)** | ***P* value** |
| **AKI stage (0, 1), n = 80,680 *** |  |  |  |  |  |
| AGs |  | **<0.001** |  |  | **0.050** |
| Without AGs | 1 |  |  | 1 |  |
| With AGs | 1.54 (1.30, 1.84) |  |  | 1.34 (1.00, 1.79) |  |
| Baseline HGB group |  | **<0.001** |  |  | 0.713 |
| >= 13 g/dL | 1 |  |  | 1 |  |
| < 13 g/dL | 1.69 (1.41, 2.02) |  |  | 0.94 (0.69, 1.29) |  |
| Baseline ALB group |  | **<0.001** |  |  | **<0.001** |
| >= 3.5 mg/dL | 1 |  |  | 1 |  |
| < 3.5 mg/dL | 3.04 (2.30, 4.03) |  |  | 2.73 (2.04, 3.65) |  |
| **AKI stage (0, 2), n = 80,399 *** |  |  |  |  |  |
| AGs |  | 0.426 |  |  | 0.954 |
| Without AGs | 1 |  |  | 1 |  |
| With AGs | 1.11 (0.86, 1.43) |  |  | 0.99 (0.66, 1.47) |  |
| Baseline HGB group |  | **0.023** |  |  | 0.506 |
| >= 13 g/dL | 1 |  |  | 1 |  |
| < 13 g/dL | 1.35 (1.04, 1.75) |  |  | 0.86 (0.55, 1.34) |  |
| Baseline ALB group |  | **<0.001** |  |  | **<0.001** |
| >= 3.5 mg/dL | 1 |  |  | 1 |  |
| < 3.5 mg/dL | 3.03 (2.03, 4.52) |  |  | 2.90 (1.91, 4.41) |  |
| **AKI stage (0, 3), n = 80,289 *** |  |  |  |  |  |
| AGs |  | 0.739 |  |  | 0.560 |
| Without AGs | 1 |  |  | 1 |  |
| With AGs | 0.94 (0.67, 1.33) |  |  | 0.85 (0.49, 1.47) |  |
| Baseline HGB group |  | 0.789 |  |  | **0.040** |
| >= 13 g/dL | 1 |  |  | 1 |  |
| < 13 g/dL | 0.95 (0.68, 1.35) |  |  | 0.55 (0.31, 0.97) |  |
| Baseline ALB group |  | **<0.001** |  |  | **<0.001** |
| >= 3.5 mg/dL | 1 |  |  | 1 |  |
| < 3.5 mg/dL | 2.74 (1.59, 4.74) |  |  | 3.04 (1.71, 5.39) |  |
| **AKD stage (0, 1), n = 728 †** |  |  |  |  |  |
| Ags |  | 0.272 |  |  | 0.236 |
| Without AGs | 1 |  |  | 1 |  |
| With AGs | 1.25 (0.84, 1.87) |  |  | 1.46 (0.78, 2.72) |  |
| Baseline HGB group |  | **0.001** |  |  | **0.029** |
| >= 13 g/dL | 1 |  |  | 1 |  |
| < 13 g/dL | 2.06 (1.33, 3.21) |  |  | 2.35 (1.09, 5.06) |  |
| Baseline ALB group |  | 0.399 |  |  | 0.713 |
| >= 3.5 mg/dL | 1 |  |  | 1 |  |
| < 3.5 mg/dL | 1.28 (0.72, 2.29) |  |  | 1.12 (0.62, 2.01) |  |
| AKI stage |  |  |  |  |  |
| 1 | 1 |  |  | 1 |  |
| 2 | 1.16 (0.75, 1.80) | 0.503 |  | 1.10 (0.59, 2.05) | 0.763 |
| 3 | 0.17 (0.04, 0.70) | **0.014** |  | 0.22 (0.03, 1.64) | 0.141 |
| **AKD stage (0, 2), n = 705 †** |  |  |  |  |  |
| AGs |  | 0.781 |  |  | 0.738 |
| Without AGs | 1 |  |  | 1 |  |
| With AGs | 1.07 (0.68, 1.67) |  |  | 0.88 (0.42, 1.84) |  |
| Baseline HGB group |  | **0.009** |  |  | 0.105 |
| >= 13 g/dL | 1 |  |  | 1 |  |
| < 13 g/dL | 1.94 (1.18, 3.17) |  |  | 2.23 (0.85, 5.90) |  |
| Baseline ALB group |  | **0.044** |  |  | 0.086 |
| >= 3.5 mg/dL | 1 |  |  | 1 |  |
| < 3.5 mg/dL | 2.50 (1.02, 6.08) |  |  | 2.20 (0.89, 5.40) |  |
| AKI stage |  |  |  |  |  |
| 1 | 1 |  |  | 1 |  |
| 2 | 2.13 (1.34, 3.39) | **0.002** |  | 1.48 (0.69, 3.14) | 0.313 |
| 3 | 0.85 (0.36, 2.01) | 0.717 |  | 0.82 (0.19, 3.56) | 0.791 |
| **AKD stage (0, 3), n = 712 †** |  |  |  |  |  |
| AGs |  | 0.263 |  |  | 0.196 |
| Without AGs | 1 |  |  | 1 |  |
| With AGs | 1.29 (0.83, 1.99) |  |  | 1.47 (0.82, 2.61) |  |
| Baseline HGB group |  | 0.758 |  |  | 0.912 |
| >= 13 g/dL | 1 |  |  | 1 |  |
| < 13 g/dL | 1.07 (0.70, 1.65) |  |  | 0.97 (0.54, 1.73) |  |
| Baseline ALB group |  | 0.721 |  |  | 0.625 |
| >= 3.5 mg/dL | 1 |  |  | 1 |  |
| < 3.5 mg/dL | 1.11 (0.63, 1.95) |  |  | 1.15 (0.65, 2.04) |  |
| AKI stage |  |  |  |  |  |
| 1 | 1 |  |  | 1 |  |
| 2 | 3.13 (1.83, 5.37) | **<0.001** |  | 2.88 (1.42, 5.85) | **0.003** |
| 3 | 6.26 (3.7, 10.56) | **<0.001** |  | 8.01 (4.08, 15.71) | **<0.001** |
| **ESRD on dialysis, n = 265 §** |  |  |  |  |  |
| AGs |  | 0.551 |  |  | NA |
| Without AGs | 1 |  |  | 1 |  |
| With AGs | 1.43 (0.44, 4.65) |  |  | NA |  |
| Baseline HGB group |  | 0.475 |  |  | NA |
| >= 13 g/dL | 1 |  |  | 1 |  |
| < 13 g/dL | 1.60 (0.44, 5.81) |  |  | NA |  |
| Baseline ALB group |  | 0.533 |  |  | 0.788 |
| >= 3.5 mg/dL | 1 |  |  | 1 |  |
| < 3.5 mg/dL | 2.01 (0.22, 17.98) |  |  | 1.35 (0.15, 12.22) |  |
| AKI stage |  |  |  |  |  |
| 1 | 1 |  |  | 1 |  |
| 2 | 1.24 (0.39, 3.91) | 0.715 |  | 0.83 (0.11, 6.22) | 0.854 |
| 3 | 0.56 (0.07, 4.55) | 0.587 |  | NA | NA |
| AKD stage |  |  |  |  |  |
| 1 | 1 |  |  | 1 |  |
| 2 | 0.86 (0.25, 2.94) | 0.810 |  | 3.90 (0.41, 37.26) | 0.237 |
| 3 | 0.54 (0.11, 2.60) | 0.442 |  | 2.82 (0.22, 36.20) | 0.425 |
| AGs, aminoglycosides (parenteral gentamicin, amikacin, and tobramycin); AKI, acute kidney injury; AKD, acute kidney disease; ALB, albumin; aHR, adjust hazard ratio; ESRD, end-stage renal disease; HGB, hemoglobin; HR, hazard ratio; NA, not available due to small sample size | | | | | |
| * model was adjusted by AGs used, baseline hemoglobin group and albumin group | | | | | |
| † model was adjusted by AGs used, baseline hemoglobin group, albumin group and AKI stage | | | | | |
| § model was adjusted by AGs used, baseline hemoglobin group, albumin group, AKI stage and AKD stage | | | | | |

| **Supplemental Table S4.** **A hazard ratio of serum HGB and ALB on kidney outcome (AKI, AKD, and ESRD on Dialysis) in AGs-used patients without prior CKD in the Cox model.** | | | | | |
| --- | --- | --- | --- | --- | --- |
|  | **HR (95% CI)** | ***P* value** |  | **aHR (95% CI)** | ***P* value** |
| **AKI stage (0, 1), n = 28,522 *** |  |  |  |  |  |
| Baseline HGB group |  | **0.009** |  |  | 0.359 |
| >= 13 g/dL | 1 |  |  | 1 |  |
| < 13 g/dL | 1.39 (1.09, 1.78) |  |  | 0.83 (0.56, 1.23) |  |
| Baseline ALB group |  | **<0.001** |  |  | **<0.001** |
| >= 3.5 mg/dL | 1 |  |  | 1 |  |
| < 3.5 mg/dL | 2.63 (1.87, 3.69) |  |  | 2.33 (1.64, 3.29) |  |
| **AKI stage (0, 2), n = 28,334 *** |  |  |  |  |  |
| Baseline HGB group |  | 0.405 |  |  | 0.849 |
| >= 13 g/dL | 1 |  |  | 1 |  |
| < 13 g/dL | 1.18 (0.80, 1.72) |  |  | 0.94 (0.52, 1.72) |  |
| Baseline ALB group |  | **0.002** |  |  | **0.012** |
| >= 3.5 mg/dL | 1 |  |  | 1 |  |
| < 3.5 mg/dL | 2.23 (1.35, 3.68) |  |  | 1.93 (1.16, 3.22) |  |
| **AKI stage (0, 3), n = 28,272 *** |  |  |  |  |  |
| Baseline HGB group |  | 0.547 |  |  | 0.114 |
| >= 13 g/dL | 1 |  |  | 1 |  |
| < 13 g/dL | 0.86 (0.51, 1.43) |  |  | 0.54 (0.25, 1.16) |  |
| Baseline ALB group |  | 0.056 |  |  | 0.077 |
| >= 3.5 mg/dL | 1 |  |  | 1 |  |
| < 3.5 mg/dL | 2.03 (0.98, 4.17) |  |  | 1.95 (0.93, 4.1) |  |
| **AKD stage (0, 1), n = 400 †** |  |  |  | 4 |  |
| Baseline HGB group |  | **0.017** |  |  | 0.086 |
| >= 13 g/dL | 1 |  |  | 1 |  |
| < 13 g/dL | 2.21 (1.15, 4.23) |  |  | 2.31 (0.89, 6.01) |  |
| Baseline ALB group |  | 0.406 |  |  | 0.666 |
| >= 3.5 mg/dL | 1 |  |  | 1 |  |
| < 3.5 mg/dL | 1.34 (0.67, 2.65) |  |  | 1.17 (0.58, 2.34) |  |
| AKI stage |  |  |  |  |  |
| 1 | 1 |  |  | 1 |  |
| 2 | 1.41 (0.80, 2.50) | 0.239 |  | 1.14 (0.54, 2.42) | 0.732 |
| 3 | 0.34 (0.08, 1.42) | 0.139 |  | 0.37 (0.05, 2.69) | 0.323 |
| **AKD stage (0, 2), n = 382 †** |  |  |  |  |  |
| Baseline HGB group |  | 0.139 |  |  | 0.182 |
| >= 13 g/dL | 1 |  |  | 1 |  |
| < 13 g/dL | 1.70 (0.84, 3.45) |  |  | 2.74 (0.62, 12.00) |  |
| Baseline ALB group |  | 0.148 |  |  | 0.282 |
| >= 3.5 mg/dL | 1 |  |  | 1 |  |
| < 3.5 mg/dL | 2.26 (0.75, 6.81) |  |  | 1.85 (0.60, 5.64) |  |
| AKI stage |  |  |  |  |  |
| 1 | 1 |  |  | 1 |  |
| 2 | 2.75 (1.49, 5.07) | **0.001** |  | 2.26 (0.89, 5.75) | 0.088 |
| 3 | 0.91 (0.27, 3.03) | 0.879 |  | 0.99 (0.13, 7.72) | 0.990 |
| **AKD stage (0, 3), n = 391 †** |  |  |  |  |  |
| Baseline HGB group |  | 0.978 |  |  | 0.379 |
| >= 13 g/dL | 1 |  |  | 1 |  |
| < 13 g/dL | 0.99 (0.56, 1.75) |  |  | 1.43 (0.64, 3.19) |  |
| Baseline ALB group |  | 0.962 |  |  | 0.855 |
| >= 3.5 mg/dL | 1 |  |  | 1 |  |
| < 3.5 mg/dL | 1.02 (0.51, 2.03) |  |  | 1.07 (0.53, 2.14) |  |
| AKI stage |  |  |  |  |  |
| 1 | 1 |  |  | 1 |  |
| 2 | 4.72 (2.46, 9.05) | **<0.001** |  | 4.35 (1.93, 9.81) | **<0.001** |
| 3 | 6.37 (3.18, 12.74) | **<0.001** |  | 7.45 (3.07, 18.07) | **<0.001** |
| **ESRD receiving dialysis, n = 160 §** |  |  |  |  |  |
| Baseline HGB group |  | 0.909 |  |  | NA |
| >= 13 g/dL | 1 |  |  | 1 |  |
| < 13 g/dL | 1.10 (0.23, 5.28) |  |  | NA |  |
| Baseline ALB group |  | 0.541 |  |  | 0.788 |
| >= 3.5 mg/dL | 1 |  |  | 1 |  |
| < 3.5 mg/dL | 1.98 (0.22, 17.74) |  |  | 1.35 (0.15, 12.22) |  |
| AKI stage |  |  |  |  |  |
| 1 | 1 |  |  | 1 |  |
| 2 | 2.17 (0.54, 8.72) | 0.276 |  | 0.83 (0.11, 6.22) | 0.854 |
| 3 | 1.13 (0.13, 10.13) | 0.912 |  | NA | NA |
| AKD stage |  |  |  |  |  |
| 1 | 1 |  |  | 1 |  |
| 2 | 0.62 (0.12, 3.20) | 0.569 |  | 3.90 (0.41, 37.26) | 0.237 |
| 3 | 0.87 (0.17, 4.47) | 0.865 |  | 2.82 (0.22, 36.20) | 0.425 |
| AGs, aminoglycosides (parenteral gentamicin, amikacin, and tobramycin); AKI, acute kidney injury; AKD, acute kidney disease; ALB, albumin; ESRD, end stage renal disease; HGB. hemoglobin; HR, hazard ratio; aHR, adjust hazard ratio; NA, not available due to small sample size | | | | | |
| * model was adjusted by baseline hemoglobin group and albumin group | | | | | |
| † model was adjusted by baseline hemoglobin group, albumin group and AKI stage | | | | | |
| § model was adjusted by baseline hemoglobin group, albumin group, AKI stage and AKD stage | | | | | |

| **Supplemental Table S5.** **A hazard ratio of serum HGB and ALB on kidney outcome (AKI, AKD, and ESRD on dialysis) in non-AGs-used patients without prior CKD in the Cox model.** | | | | | |
| --- | --- | --- | --- | --- | --- |
|  | **HR (95% CI)** | ***P* value** |  | **aHR (95% CI)** | ***P* value** |
| **AKI stage (0, 1), n = 36,462 *** |  |  |  |  |  |
| Baseline HGB group |  | **0.002** |  |  | 0.714 |
| >= 13 g/dL | 1 |  |  | 1 |  |
| < 13 g/dL | 1.56 (1.18, 2.05) |  |  | 1.10 (0.66, 1.86) |  |
| Baseline ALB group |  | **<0.001** |  |  | **<0.001** |
| >= 3.5 mg/dL | 1 |  |  | 1 |  |
| < 3.5 mg/dL | 3.84 (2.35, 6.30) |  |  | 3.71 (2.21, 6.21) |  |
| **AKI stage (0, 2), n = 36,370 *** |  |  |  |  |  |
| Baseline HGB group |  | 0.124 |  |  | 0.310 |
| >= 13 g/dL | 1 |  |  | 1 |  |
| < 13 g/dL | 1.34 (0.92, 1.93) |  |  | 0.72 (0.38, 1.36) |  |
| Baseline ALB group |  | **<0.001** |  |  | **<0.001** |
| >= 3.5 mg/dL | 1 |  |  | 1 |  |
| < 3.5 mg/dL | 5.06 (2.59, 9.87) |  |  | 5.55 (2.76, 11.14) |  |
| **AKI stage (0, 3), n = 36,324 *** |  |  |  |  |  |
| Baseline HGB group |  | 0.873 |  |  | 0.140 |
| >= 13 g/dL | 1 |  |  | 1 |  |
| < 13 g/dL | 0.96 (0.59, 1.57) |  |  | 0.54 (0.24, 1.22) |  |
| Baseline ALB group |  | **0.001** |  |  | **<0.001** |
| >= 3.5 mg/dL | 1 |  |  | 1 |  |
| < 3.5 mg/dL | 4.33 (1.87, 10.02) |  |  | 5.18 (2.16, 12.45) |  |
| **AKD stage (0, 1), n = 321 †** |  |  |  |  |  |
| Baseline HGB group |  | 0.058 |  |  | 0.174 |
| >= 13 g/dL | 1 |  |  | 1 |  |
| < 13 g/dL | 1.86 (0.98, 3.51) |  |  | 2.44 (0.67, 8.81) |  |
| Baseline ALB group |  | 0.821 |  |  | 0.995 |
| >= 3.5 mg/dL | 1 |  |  | 1 |  |
| < 3.5 mg/dL | 1.13 (0.38, 3.39) |  |  | 1.00 (0.33, 3.02) |  |
| AKI stage |  |  |  |  |  |
| 1 | 1 |  |  | 1 |  |
| 2 | 0.95 (0.48, 1.88) | 0.892 |  | 1.04 (0.35, 3.11) | 0.947 |
| 3 | NA | NA |  | NA | NA |
| **AKD stage (0, 2), n = 315 †** |  |  |  |  |  |
| Baseline HGB group |  | **0.025** |  |  | 0.417 |
| >= 13 g/dL | 1 |  |  | 1 |  |
| < 13 g/dL | 2.26 (1.11, 4.59) |  |  | 1.73 (0.46, 6.51) |  |
| Baseline ALB group |  | 0.166 |  |  | 0.189 |
| >= 3.5 mg/dL | 1 |  |  | 1 |  |
| < 3.5 mg/dL | 2.93 (0.64, 13.35) |  |  | 2.78 (0.61, 12.76) |  |
| AKI stage |  |  |  |  |  |
| 1 | 1 |  |  | 1 |  |
| 2 | 1.59 (0.77, 3.27) | 0.209 |  | 0.71 (0.19, 2.68) | 0.615 |
| 3 | 0.79 (0.23, 2.68) | 0.702 |  | 0.66 (0.08, 5.41) | 0.702 |
| **AKD stage (0, 3), n = 314 †** |  |  |  |  |  |
| Baseline HGB group |  | 0.936 |  |  | 0.092 |
| >= 13 g/dL | 1 |  |  | 1 |  |
| < 13 g/dL | 1.03 (0.51, 2.07) |  |  | 0.44 (0.17, 1.14) |  |
| Baseline ALB group |  | 0.604 |  |  | 0.480 |
| >= 3.5 mg/dL | 1 |  |  | 1 |  |
| < 3.5 mg/dL | 1.30 (0.49, 3.45) |  |  | 1.43 (0.53, 3.89) |  |
| AKI stage |  |  |  |  |  |
| 1 | 1 |  |  | 1 |  |
| 2 | 1.61 (0.60, 4.33) | 0.344 |  | 0.72 (0.14, 3.70) | 0.690 |
| 3 | 6.32 (2.79, 14.32) | **<0.001** |  | 8.19 (2.79, 24.02) | **<0.001** |
| **ESRD receiving dialysis, n = 104 §** |  |  |  |  |  |
| Baseline HGB group |  | 0.405 |  |  | NA |
| >= 13 g/dL | 1 |  |  | 1 |  |
| < 13 g/dL | 2.62 (0.27, 25.24) |  |  | NA |  |
| Baseline ALB group |  | NA |  |  | NA |
| >= 3.5 mg/dL | 1 |  |  | 1 |  |
| < 3.5 mg/dL | NA |  |  | NA |  |
| AKI stage |  |  |  |  |  |
| 1 | 1 |  |  | 1 |  |
| 2 | 0.41 (0.04, 3.98) | 0.445 |  | NA | NA |
| 3 | NA | NA |  | NA | NA |
| AKD stage |  |  |  |  |  |
| 1 | 1 |  |  | 1 |  |
| 2 | 1.40 (0.20, 9.91) | 0.739 |  | NA | NA |
| 3 | NA | NA |  | NA | NA |
| AGs, aminoglycosides (parenteral gentamicin, amikacin, and tobramycin); AKI, acute kidney injury; AKD, acute kidney disease; ALB, albumin; ESRD, end stage renal disease; HGB, hemoglobin; HR, hazard ratio; aHR, adjust hazard ratio; NA, not available due to small sample size | | | | | |
| * model was adjusted by baseline hemoglobin group and albumin group | | | | | |
| † model was adjusted by baseline hemoglobin group, albumin group and AKI stage | | | | | |
| § model was adjusted by baseline hemoglobin group, albumin group, AKI stage and AKD stage | | | | | |
